# *Akkermansia muciniphila* as a Model Case for the Development of an Improved Quantitative RPA Microbiome Assay

**DOI:** 10.3389/fcimb.2018.00237

**Published:** 2018-07-12

**Authors:** Heather J. Goux, Dimple Chavan, Mary Crum, Katerina Kourentzi, Richard C. Willson

**Affiliations:** ^1^Department of Biology and Biochemistry, University of Houston, Houston, TX, United States; ^2^Department of Chemical and Biomolecular Engineering, University of Houston, Houston, TX, United States; ^3^Tecnológico de Monterrey-ITESM Campus Monterrey, Monterrey, Mexico

**Keywords:** RPA, gut microbiome, *Akkermansia muciniphila*, bacterial quantification, point-of-need

## Abstract

Changes in the population levels of specific bacterial species within the gut microbiome have been linked to a variety of illnesses. Most assays that determine the relative abundance of specific taxa are based on amplification and sequencing of stable phylogenetic gene regions. Such lab-based analysis requires pre-analytical sample preservation and storage that have been shown to introduce biases in the characterization of microbial profiles. Recombinase polymerase amplification (RPA) is an isothermal nucleic acid amplification method that employs commercially available, easy-to-use freeze-dried enzyme pellets that can be used to analyze specimens rapidly in the field or clinic, using a portable fluorometer. Immediate analysis of diverse bacterial communities can lead to a more accurate quantification of relative bacterial abundance. In this study, we discovered that universal bacterial 16S ribosomal DNA primers give false-positive signals in RPA analysis because manufacturing host *Escherichia coli* DNA is present in the RPA reagents. The manufacturer of RPA reagents advises against developing an RPA assay that detects the presence of *E. coli* due to the presence of contaminating *E. coli* DNA in the reaction buffer (www.twistdx.co.uk/). We, therefore, explored four strategies to deplete or fragment extraneous DNA in RPA reagents while preserving enzyme activity: metal-chelate affinity chromatography, sonication, DNA cleavage using methylation-dependent restriction endonucleases, and DNA depletion using anti-DNA antibodies. Removing DNA with anti-DNA antibodies enabled the development of a quantitative RPA microbiome assay capable of determining the relative abundance of the physiologically-important bacterium *Akkermansia muciniphila* in human feces.

## Introduction

The human intestinal microbiome contains ≥10^14^ bacteria representing over 400 species (Ott et al., 2004). Recent publications have suggested that the composition of the gut microbiota is significantly associated with health and disease (Shreiner et al., 2015; Lloyd-Price et al., 2016; Lynch and Pedersen, 2016; Duvallet et al., 2017). Relatively small changes in bacterial levels from key taxonomic groups have been linked to a wide range of illnesses, including inflammatory bowel disease, Crohn's disease, colon cancer, and hyperglycemia (Watterlot et al., 2008; Kang et al., 2013; Scher et al., 2013; Scheperjans et al., 2015; Schneeberger et al., 2015; Dao et al., 2016; Rosa et al., 2017; Wong et al., 2017). The use of pro- or prebiotics or fecal microbiota transplantation have been shown to alter the microbial profile of the gut, in some cases improving health (Muegge, 2011; Petrof et al., 2013; Colman and Rubin, 2014; Cui et al., 2015; Plovier et al., 2016; Routy et al., 2018). Development of a method to monitor the abundance of beneficial microorganisms within the complex milieu of the gut microbiome is therefore of considerable interest.

A variety of techniques are available for analyzing microbiota composition, including small-subunit ribosomal RNA (16S rRNA) gene sequencing, whole-metagenome shotgun sequencing, quantitative polymerase chain reaction assays, and microbial culture (Morgan and Huttenhower, 2012). All prokaryotes harbor a 16S rRNA gene, which includes both conserved sequences and species-specific hypervariable regions. Well-developed databases (e.g., GreenGenes, Ribosomal Database Project, and Silva) are available to classify 16S rRNA sequence data at high taxonomic resolution for use in microbial population profiling. Sequencing short 16S rRNA gene segments often is more cost-effective than sequencing the entire metagenome and thus enables cohort studies large enough to identify statistically significant correlations with disease states (Morgan and Huttenhower, 2012; Hermann-Bank et al., 2013; Robinson et al., 2016). Although 16S rRNA sequencing is currently the favored tool for the detection of bacterial biomarkers, qPCR is faster, cheaper, and easier to interpret, making it the preferred method for biomarker validation (Watterlot et al., 2008; Kostic et al., 2012; Kang et al., 2013; Scher et al., 2013; Zhang et al., 2016). In addition, qPCR often enables more accurate quantification of specific species than 16S rRNA sequencing (Hermann-Bank et al., 2013). However, PCR and 16S detection methods can be subject to biases associated with pre-analytical sample preservation, storage, and DNA extraction (Robinson et al., 2016), leading to inaccuracies in fecal bacterial quantification. Reducing the amount of sample handling and eliminating sample storage can lead to a more accurate estimation of bacterial abundance within the gut.

Recombinase polymerase amplification (RPA) is an isothermal amplification nucleic acid detection method suitable for analysis of samples at the point of need (Euler et al., 2012a; Abd El Wahed et al., 2013, 2015; Rosser et al., 2015; Bonney et al., 2017; Kim and Lee, 2017). Rather than heat denaturation, RPA uses recombinases (*E. coli* RecA) to form a complex with signal stranded oligonucleotides (30–35 nt primers) and single strand binding proteins (SSBs) assist site-specific D-loop strand invasion (Piepenburg et al., 2006) of dsDNA. At a constant temperature of 37–42°C, *Sau* (*Staphylococcus aureus)* DNA polymerase performs primer-extension to generate a new strand of DNA. Much like PCR, the newly generated product goes on to become the template for future rounds of amplification. Incorporation of a cleavable self-quenched exo-probe or SYBR Green dye allows for real-time fluorescence monitoring of RPA assays, thus enabling quantitative analysis typically within 10–15 min (Crannell et al., 2014, 2015; Kim and Lee, 2016, 2017; Moore and Jaykus, 2017). As RPA utilizes freeze-dried, reaction-ready enzyme pellets (manufactured by TwistDx, Inc.) and a portable fluorometer, the technique can be readily adapted to field applications (Abd El Wahed et al., 2015). Immediate field-based analysis of diverse bacterial communities can lead to a more accurate quantification of relative bacterial abundance.

Although RPA can provide valuable data for quantifying bacterial taxa within the gut microbiome, transitioning from an exploratory 16S rRNA–based sequencing study to a PCR/RPA-based confirmatory study can be complicated by differences in the way taxonomic abundance is defined (Ott et al., 2004; Morgan and Huttenhower, 2012; Gloor et al., 2017). In real-time RPA, bacteria are quantified according to a standard curve with abundance defined in terms of gene copies per unit volume (Euler et al., 2012b; Crannell et al., 2014, 2015; Kim and Lee, 2017). By contrast, in 16S rRNA–based sequencing, bacterial abundance is estimated based on the fraction of total observed 16S rRNA sequences assignable to a particular taxonomic group (with the complication that the number of 16S rRNA genes per genome can vary; Robinson et al., 2016). Thus, correlations based on 16S rRNA–based sequencing are relative rather than absolute (Ott et al., 2004); as such, subsequent quantitative PCR/RPA data should ideally be reported in terms of relative abundance. One way this has been achieved in gut bacterial PCR studies is by calculating the ratio of group-specific to total 16S rRNA abundance (Hermann-Bank et al., 2013; Brukner et al., 2015; Zhang et al., 2016). This method can be easily adapted to RPA to quantify target organisms.

The importance of accurate quantification of specific organisms within the gut microbiome can be illustrated by the case of diabetes, which affected 9.4% of Americans (30.3 million individuals) in 2015 (Centers for Disease Control and Prevention, 2017). *Akkermansia muciniphila* is a gram-negative, anaerobic, mucin-degrading bacterium commonly found in high abundance in the human gut. Low abundance of *A. muciniphila* has been linked to hyperglycemia, glucose intolerance, obesity, and type 2 diabetes (Everard et al., 2011, 2013; Louis et al., 2016; Yassour et al., 2016). *A. muciniphila* is also a key producer of short-chain fatty acids in the gut, which have been shown to inhibit inflammation and aid in metabolic dysregulation (Dao et al., 2016). In several mouse studies, administering *A. muciniphila* to diabetic subjects improved their metabolic functions and aided in weight loss (Greer et al., 2016; Plovier et al., 2016; Hänninen et al., 2017). The medical community may soon need an inexpensive screening tool for identifying individuals with lower fecal *A. muciniphila* abundance that could benefit from therapeutic intervention. We, therefore, chose *A. muciniphila* as a model organism in developing a quantitative RPA microbiome assay.

During assay development, we found that non-specific amplification occurred using 16S rRNA universal primer pairs in the absence of added DNA template, reducing the accuracy of total bacterial load quantification. Amplification of residual *Escherichia coli* production-host DNA contained in the recombinant RPA reagents was identified as the confounding factor and was confirmed by Sanger sequencing. Relative *A. muciniphila* abundance is calculated from the ratio of *A. muciniphila* to total 16S rRNA abundance. Therefore, an inability to quantify the total bacterial load precludes accurate estimation of the relative abundance of *A. muciniphila*. To resolve this issue, we explored four strategies to deplete or destroy extraneous *E. coli* DNA while preserving RPA reagent functionality: (1) metal-chelate affinity capture; (2) sonication; (3) methylation-dependent restriction endonuclease digestion; and (4) DNA capture/removal using anti-DNA antibodies. We show that removal of interfering DNA using anti-DNA antibodies was the most effective strategy. Using RPA reagents treated with anti-DNA antibodies, we then constructed a quantitative total bacterial standard curve and determined the relative abundance of *A. muciniphila* in a human fecal sample to demonstrate the application of the quantitative RPA microbiome assay.

## Materials and methods

### Design of bacteria-specific primers

It is suggested that RPA primers be 30–35 nt long for optimal amplification of the template. Only 9% of previously published RPA primers are below 30 nt in length (Daher et al., 2016). RPA was attempted with widely-used 16S universal primers (primer set 2; Table 1) which resulted in a significant delay in amplification. We, therefore, undertook to design our own bacteria-specific primers 28–35 nt in length and with GC content of 30–70%. Highly-conserved regions within the 16S rRNA gene were chosen as the targets for bacteria-specific RPA primers. When designing a 16S gene specific universal primer, it is likely that large increases in primer length would lead to an increase in the number of mismatches. However, guidelines for RPA primer design are not as stringent as those for the design of PCR primers. Longer RPA primers tolerate some additional mismatches while still achieving adequate amplification (Daher et al., 2015). The National Center for Biotechnology Information BLAST tool was used to test each primer's potential match to all publicly-available bacteria genome sequences. A match was defined for this purpose as less than six total mismatches and less than four mismatches in the last six 3′ nucleotides of either the primer or the target sequence. Primer sequences complementary to human DNA were identified using BLAST and excluded in order to reduce the probability of amplifying human DNA. The free-energy calculation function of the OligoAnalyzer tool (http://www.idtdna.com/analyzer/Applications/OligoAnalyzer) was used to assess the potential formation of secondary structures (primer dimers and hairpins) and avoid inter- or intra-molecular interactions with a ΔG of <−6 kcal.mol^−1^.

**Table 1:** General bacteria- and *Akkermansia muciniphila*–specific primer pairs.

**Primer set**	**Specificity**	**Sequence (5′-3′)**	**Length (nt)**	**References**
**PRIMER SET 1**				
Fwd	Bacteria	att gaa gag ttt gat cat ggc tca gat t	28	This work
Rvs		ccg tgt ctc agt tcc agt gtg gct ggt c	28	
**PRIMER SET 2**				
P338f	Bacteria	act cct acg gga ggc agc ag	20	Muyzer et al., 1993; Schneeberger et al., 2015
P518r		att acc gcg gct gct gg	17	
**PRIMER SET 3**				
Fwd	*A. muciniphila*	gcg tag gct gtt tcg taa gtc gtg tgt gaa ag	32	This work
Rvs		gag tgt tcc cga tat cta cgc att tca	30	
**PRIMER SET 4**				
Fwd	*A. muciniphila*	cag cac gtg aag gtg ggg	18	Collado et al., 2007; Schneeberger et al., 2015; Guo et al., 2016
Rvs		cct tgg ggt tgg ctt cag at	20	
**PRIMER SET 5**				
515f	Bacteria	gtg cca gcm gcc gcg gta a	18	Caporaso et al., 2011
806r		gga cta chv ggg twt cta at	20	

During *A. muciniphila* specific primer design, two different *A. muciniphila* 16S rRNA gene reference sequences (American Type Culture Collection [ATCC] strain BAA-835; accession number NR_074436.1 and accession number NR_042817.1) were downloaded from GenBank (http://www.ncbi.nlm.nih.gov/genbank) and aligned using SeaView (Gascuel et al., 2010) to identify species-specific conserved sequences within the 16S rRNA gene variable region. The newly-developed *A. muciniphila* specific primers (primer set 3, Table 1) were tested for species-specificity using an NCBI BLAST search tool to confirm zero mismatches to all 39 *A. muciniphila* genomes in the GenBank reference genome database. The NCBI BLAST tool was also used to test each primer's off-target specificity to all publicly-available bacteria genome sequences outside of *A. muciniphila*. The reverse primer was found to be 100% matched to two off-target organisms, *Haloferula rosa* and *Luteolibacter algae* (Verrucomicrobia phylum)*. Luteolibacter algae* and *H. rosa* are two species commonly found in marine environments (Yoon et al., 2008; He et al., 2017) and are not known to inhabit the human gut in significant abundance. Using data from the U.S. NIH Human Microbiome Project and the search engine EZ Bio Cloud (https://www.ezbiocloud.net/resources/human_microbiome) we found no presence of *L. algae* and *H. rosa* in stool. The *Akkermansia* genus dominates the Verrucomicrobia population found in the human gut (Dubourg et al., 2013). When found in the gut, the abundance of these species is not enough to significantly affect the calculation of *A. muciniphila* abundance (0–5% of total reads; Zhang et al., 2015).

### Genomic DNA standards

*E. coli* strain 1532 (ATCC 35218) was streaked onto a 5% sheep's blood agar plate (Becton, Dickinson and Company; Franklin Lakes, NJ) and incubated for 12 h at 37°C. Genomic DNA (gDNA) was isolated from *E. coli* cultures using an UltraClean Microbial DNA Isolation kit (Mo Bio Laboratories; Carlsbad, CA). The absorbance of isolated *E. coli* and commercially obtained *A. muciniphila* gDNA (strain ATCC BAA-835) was measured at 230, 260, and 280 nm using a Nanodrop 1000 (NanoDrop Instruments, Wilmington, DE). The 260/280 and 260/230 absorbance ratios were ≥2.0, confirming gDNA purity. The gDNA concentration, expressed as genome copies per μL, was calculated using the absorbance at 260 nm, the extinction coefficient of double-stranded DNA (0.020 μg^−1^ mL cm^−1^), the average molar weight of a DNA base pair (650 g mol^−1^), the size of each reference strain's genome (*E. coli* ATCC 35218, 4.64 Mbp; *A. muciniphila* ATCC BAA-835, 2.66 Mbp), and Avogadro's number. gDNA aliquots (10 μL) were stored at −20°C and used as RPA standards. Ten-fold serial dilutions of both *E. coli* and *A. muciniphila* gDNA were made using nuclease-free deionized water. Finally, 2 or 5 μL of template (100–10^7^ copies of *E. coli* or *A. muciniphila* gDNA) were added to quantitative RPA and PCR reactions.

### Quantitative real-time RPA

Real-time RPA reactions were performed using a TwistAmp Basic kit (TwistDx, Cambridge, UK, TABAS03KIT) with primers purchased from Integrated DNA Technologies (Coralville, IA). Master mix (45.5 μL) containing 420 nM primers (primer pair set 1 or 2, Table 1), SYBR green dye I (ThermoFisher Scientific, Product #S7567; 45,500-fold dilution of stock concentration), and TwistAmp rehydration buffer was prepared and distributed into TwistAmp Basic reaction tubes. Next, 2 or 5 μL of template (100–10^6^ copies of *E. coli* or *A. muciniphila* gDNA standard) and 2.5 μL of 280 mM magnesium acetate (MgAc) were added to the reaction mix to initiate the amplification reaction. Tubes were then placed into an Agilent MxPro 3005 real-time PCR machine (Agilent Technologies, Santa Clara, CA). Fluorescence (excitation, 497 nm; detection, 520 nm) was measured every 15 s for 60 min at 37°C.

### Strategies for removing extraneous DNA from RPA reagents

#### Strategy 1: removing DNA using metal-chelate affinity capture

Chelating Sepharose fast-flow beads (25 μL; catalog no. 17057502, GE Healthcare; cross-linked 6% agarose functionalized with iminodiacetic acid groups) were charged according to the manufacturer's protocol with Ni^2+^ ions to enable interaction with aromatic DNA base nitrogen atoms (Murphy et al., 2003; Cano et al., 2005) and then resuspended in 70 μL of 60 mM Tris buffer (pH 7) containing 1 M NaCl. Four TwistDx TwistAmp RPA reaction pellets were rehydrated in 70 μL of TwistDx Rehydration Buffer. The bead and RPA reagent suspensions were combined and mixed on a rotator at end-over-end at 3 rpm for 2 h at 4°C. The mixture was then centrifuged at 5,500 x*g* for 2 min to remove the beads, and the supernatant was aliquoted into four PCR tubes (35 μL per tube). Master mix (47.5 μL) containing 505 μM primers and SYBR green dye (47,500-fold dilution of stock solution) was added to each tube along with 2 μL of *E. coli* gDNA standard (100 copies per μL). Next, 2.5 μL of 280 mM MgAc was added to the PCR tubes, which were then placed into an Agilent MxPro 3005 Real-Time PCR machine and amplified at 37°C.

#### Strategy 2: DNA shearing by sonication

Individual TwistAmp RPA Reaction pellets were reconstituted with 29.5 μL of TwistDx Rehydration Buffer, placed on ice, and sonicated at a amplitude of 40 for 10 cycles (3 s on, 7 s off) using an ultrasonic homogenizer (Model 150V/T, Biologics Inc.). Next, 29.5 μL of the sonicated suspension and 16 μL of master mix (containing 1.5 μM primer set 1 and 16,000-fold dilution of SYBR green dye) were pipetted into new PCR tubes. Template (2 μL; 100 *E. coli* gDNA copies per μL) and 2.5 μL of 280 mM MgAc were added to initiate RPA, and the tubes were placed into an Agilent MxPro 3005 real-time PCR machine for amplification.

#### Strategy 3: methylation-dependent endonuclease digestion

RPA pellets were suspended in 29.5 μL of Rehydration Buffer with 0–140 units of DpnI restriction endonuclease (New England Biolabs, product #R0176S) and incubated for 15 or 60 min at 37°C, with or without 14 mM MgAc, to cleave methylated *E. coli* gDNA at Gm_6_ATC restriction sites. After digestion, tubes with treated pellet solution were mixed with 47.5 μL of master mix containing 505 μM primers and SYBR green dye (47,500-fold dilution of stock solution). Finally, 2 μL (100 copies) of *E. coli* gDNA standard and 2.5 μL of 280 mM MgAc were added to the tube caps and spun down in a microcentrifuge to simultaneously initiate the RPA reactions.

#### Strategy 4: DNA depletion using anti-DNA antibodies

Anti-dsDNA antibodies coupled to amine-modified magnetic particles were prepared as follows. Nine hundred microliters of 55.6 μg mL^−1^ anti-dsDNA antibody (#ab27156, Abcam) in 100 mM sodium acetate buffer (pH 5.4) was added to 45 μL of 0.1 M NaIO_4_. After 30 min of incubation at room temperature (RT), oxidized antibodies were concentrated using 100-kDa Amicon Ultra centrifugal filters (Millipore, Billerica, MA, USA) and diluted to 500 μL at 100 μg mL^−1^ in 200 mM sodium carbonate buffer (pH 9.6). Next, 3.1-μm Promag amine microspheres (200 μL; 1 × 10^8^ particles; Bangs Laboratories Inc.) were washed and resuspended in 500 μL of 200 mM sodium carbonate buffer (pH 9.6) and then added to the oxidized antibodies. After incubation at RT for 2 h, 15 μL of 5 M NaCNBH_3_, 1 M NaOH was added to the reaction and incubated for an additional 30 min at RT. Next, 75 μL of 1 M hydroxylamine was added, and the mixture was incubated for 30 min. The antibody-functionalized magnetic particles were washed 3 times and stored at 4°C in phosphate-buffered saline (PBS) (final concentration, 1 × 10^5^ particles per μL).

To remove extraneous DNA, TwistAmp Basic kit freeze-dried pellets (TwistDx) were reconstituted in 29.5 μL of TwistDx Rehydration Buffer and transferred to a low-binding microcentrifuge tube containing 2 × 10^5^ antibody-functionalized magnetic beads in 2 μL of buffer. The microcentrifuge tube was placed on a rotator and incubated for 30 min at 4°C, after which the beads were removed using a magnet, and the solution was transferred to new tubes in 29.5-μL aliquots. Master mix (47.5 μL) containing 505 μM primers and SYBR green dye (47,500-fold dilution of the stock solution) was added to each tube along with *E. coli* gDNA (100 genome copies per μL) in 2 μL. Finally, 2.5 μL of 280 mM MgAc was added to the tube caps and spun in a microcentrifuge to simultaneously initiate RPA.

### qPCR assays

Real-time PCR standard curves were prepared with 10-fold serial dilutions of gDNA standards (*E. coli* ATCC 35218 or *A. muciniphila* ATCC BAA-835) using previously-validated primer pairs (16S rRNA bacteria-specific primer set 2, or *A. muciniphila* specific primer set 4, Table 1). qPCR was performed in 20-μL reaction volumes containing 550 nM primers (1.1 μL of 10 μM), a 1 × concentration of Brilliant III Ultra-Fast SYBR green qPCR master mix (10 μL of 2 × commercial stock conc.; Agilent Technologies), and 2 or 5 μL of template. Amplification curves, baselines, and threshold cycles were calculated using Agilent MxPro 3005P software as described below.

### Threshold time and cycle parameters for RPA and qPCR reactions

MxPro software (Agilent Technologies) was used to normalize baseline fluorescence and calculate RPA threshold times from raw fluorescence data. Using a linear least mean squares algorithm, the baseline function was calculated by fitting the raw fluorescence in the first 3 min of the reaction to a first-order function. Baseline-corrected fluorescence was obtained by subtracting the baseline from the raw fluorescence to plot an amplification curve. For each assay, threshold fluorescence was defined as the point at which the fluorescence exceeded the average baseline-corrected fluorescence by three standard deviations, located in the exponential region of the amplification curve. The threshold time or cycle was calculated as the time or cycle at which the reaction met the threshold fluorescence. For each assay, average threshold cycle (*n* = 3) was plotted against copies of gDNA per reaction on semi-logarithmic axes to generate a regression line.

### Determining total bacterial and *A. muciniphila* abundance

An anonymized human fecal sample from a deceased Caucasian female with a medical history of hypoglycemia and hypothyroidism was obtained from Analytical Biological Services Inc. Three micrograms of DNA (15 ng/μL) was isolated from 220 mg of the fecal sample using QIAamp DNA Stool mini kit (catalog no. 51504, Qiagen). Nucleic acid purity was confirmed by a 260 nm/280 nm absorbance ratio of 1.83 using Nanodrop™. Either 3 or 7.5 ng of isolated DNA (2- or 5-μL of a 10-fold dilution of the stock concentration) was run as the template in quantitative RPA and PCR assays. Average threshold time (RPA) or cycle (PCR) and relative standard curves were used to determine the number of *E. coli* or *A. muciniphila* gDNA copies per 15 ng of the isolated DNA. Finally, the relative *A. muciniphila* abundance was calculated as the ratio of *A. muciniphila* gDNA copies per μL to the number of bacterial gDNA copies per μL.

For both *A. muciniphila* (ATCC BAA-835) and *E. coli* (ATCC 35218) gDNA standards, the 16S rRNA gene sequences from the GenBank (accession nos. NR_074436.1 and EF436579, respectively) were aligned to the complete genome sequences of *A. muciniphila* (accession no. NC_010655.1) and *E. coli* (accession NZ_KK583188.1) using the NCBI BLAST. This resulted in three and seven matches (100% in identity and composition) for *A. muciniphila* and *E. coli*, respectively. Thus, to calculate the 16S rRNA gene copies for each standard, the genomic DNA copies were multiplied by the number of 16rRNA gene copies per genome (3 for *A. muciniphila* and 7 for *E. coli*).

### Sequencing-based detection

The composition of the fecal microbiome was independently characterized using 16S rRNA sequencing. A QIAamp DNA Stool mini kit was used to isolate DNA from the fecal sample. SeqWright Genomic Services (Houston, TX) generated and validated a library of 300-bp amplicons (MiSeq Reagent kit v3, MS-102-3001) using the extracted DNA and a universal primer pair (primer set 5; Table 1) spanning the 16S rRNA V4 region (Caporaso et al., 2011). Sequencing was performed on an Illumina MiSeq instrument with 250-bp paired-end reads. Reads were then uploaded to the Sequencing Read Archive (BioProjectID: PRJNA472995) and Illumina Basespace Sequencing Hub for sequencing analysis. Kraken Metagenomics software was used to assign taxonomic labels to each read using a k-mer–based algorithm. The relative abundance of *A. muciniphila* was then calculated by comparing the number of reads classified as *A. muciniphila* to the total number of bacterial reads.

## Results

### Identification of *Escherichia coli* DNA contamination in RPA reagents

With unmodified RPA reagents, amplification was observed with primer set 1 in reactions containing 100–1,000,000 copies of *E. coli* gDNA in 10.3 min essentially independent of initial template concentration (standard deviation: 60 s, range: 10–11 min); no-template controls also showed amplification in 9.5 min (*n* = 1) (Figure S1). To confirm that the newly designed primer pair (primer set 1) was bacteria-specific, the amplification curves generated using primer set 1 were compared to amplification curves generated using the previously published and validated universal primer set 2 (Table 1, Figure S1). Amplification was observed with primer set 2 in reactions containing 100–1,000,000 copies of *E. coli* gDNA in 24.2 min (standard deviation: 150 s, range: 23.2–25.8 min), essentially independent of template concentration; the no-template control also showed amplification in 24.8 min (*n* = 1). A difference in primer length is the cause of the large jump in threshold time that is observed when no-template control reactions are tested with the two universal primer pairs. The manufacturer of the RPA reagent (TwistDx) recommends that RPA primers be 30–35 nt long. When RPA reactions are tested with PCR-size primers (15–22 nt in length), reactions will take longer to achieve threshold fluorescence than when reactions are tested with primers of the recommended length.

Amplicons generated from the no-template reaction with primer set 1 were purified using a QIAquick PCR Purification kit. To determine if primer dimers were contributing to unintended amplification, the purified RPA product was analyzed by gel electrophoresis to show the expected ~330-bp single band. To determine the phylogenic origin of the amplicon, the product was Sanger sequenced (Genewiz, Houston, TX) and the sequencing traces analyzed with Sequence Scanner 2 (Applied Biosystems, Foster City, CA) to generate a consensus sequence. The consensus sequence was aligned against NCBI's 16S ribosomal RNA database using nBLAST to show the highest similarity (99–100% identity) when aligned with a portion of the 16S rRNA gene in *E. coli*. Information provided by the manufacturer indicated that essential enzymes in RPA reagents are produced in an *E. coli* expression host. We, therefore, hypothesized that DNA from the *E. coli* vector is present in the RPA reaction pellets and amplified when 16S rRNA universal primers are used.

### Strategies for removing extraneous DNA from RPA reagents

#### Removing DNA using metal-chelate affinity capture

As the purines of single-stranded nucleic acids contain an imidazole ring similar to that of the histidines recognized in “His_6_-tagging,” they exhibit a strong affinity for chelated transition metal ions (Murphy et al., 2003; Cano et al., 2005). Thus, we hypothesized that Ni-IDA immobilized metal affinity chromatography (IMAC) agarose beads would be an effective means of selectively binding and removing extraneous DNA from RPA reagents.

RPA reaction pellets were first reconstituted in rehydration buffer and then incubated with Ni^+2^-loaded IDA sepharose particles, as described in section Materials and Methods. The particles were removed by centrifugation, and the resulting supernatant was tested using primer set 1 in real-time RPA reactions spiked with 100 copies of *E. coli* gDNA (data not shown). Amplification curves generated using Ni-treated, and untreated reagents were indistinguishable, indicating that: (1) there was no significant decline in reaction efficiency after incubation with the IMAC resin; (2) RPA proteins exhibited low nonspecific (or His-tag) binding to Ni-IDA sepharose beads; and (3) little, if any, DNA was removed during the treatment of RPA pellets. Given our previous finding that Ni-IDA affinity is specific to single-stranded nucleic acids with exposed purine bases (Murphy et al., 2003; Cano et al., 2005), this result suggests that the majority of the residual DNA in the RPA reagents is double-stranded.

#### DNA shearing by sonication

Sonication is widely used in next-generation sequencing protocols to shear genomic DNA into fragments of as little as 150–200 bp. Gel electrophoresis analysis of the product generated from primer set 2, and no-template control reactions showed a single band, ~300 bp in length. Thus, we hypothesized that sonicating the reaction pellet would render the contaminating DNA un-amplifiable in downstream RPA reactions. No-template reactions with sonicated reagents reached the threshold fluorescence, on average, 60 s earlier than reactions with untreated reagents (Figure S2). Thus, we concluded that: (1) RPA reagents are tolerant of the degree of sonication applied, and (2) sonicating RPA pellets was not sufficient to shear extraneous DNA to such a degree as to impair amplification.

This result is consistent with previous studies which showed increases in amplification efficiency when samples are sonicated before PCR (Golenberg et al., 1996; Veal et al., 2012). Fragmenting genomic DNA into ~1 kb segments suggests facilitation of DNA dehybridization in GC-rich regions for quicker binding of polymerase at recognition sites and enhanced strand amplification.

#### Methylation-dependent endonuclease digestion

DpnI is a methylation-dependent restriction endonuclease that cleaves DNA prepared from *E. coli* dam^+^ strains but not PCR-amplified DNA (Glickman, 1980; Barnes et al., 2014). The DpnI cleavage site (5′-Gm_6_ATC) was found to occur twice within the region of predicted amplification in the *E. coli* 16S rRNA gene (Figure S3). Thus, pre-treating RPA reagents with DpnI restriction enzymes should digest and render the extraneous DNA un-amplifiable.

Laborious removal of DpnI after incubation and prior to initiation of the RPA reaction likely would be unnecessary. As RPA reactions are isothermal, they commence immediately upon mixing of the reagents. This continuous amplification leads to prompt generation of synthetic products (Piepenburg et al., 2006). If sample (reaction template) and primers are simultaneously added to the DpnI-treated pellet, a portion of the sample DNA is likely to evade DpnI cleavage, allowing it to be amplified during the first round of RPA. The resulting unmethylated synthetic products are impervious to DpnI enzymatic cleavage and are available to act as a template in subsequent rounds of amplification.

**Figure 1:**
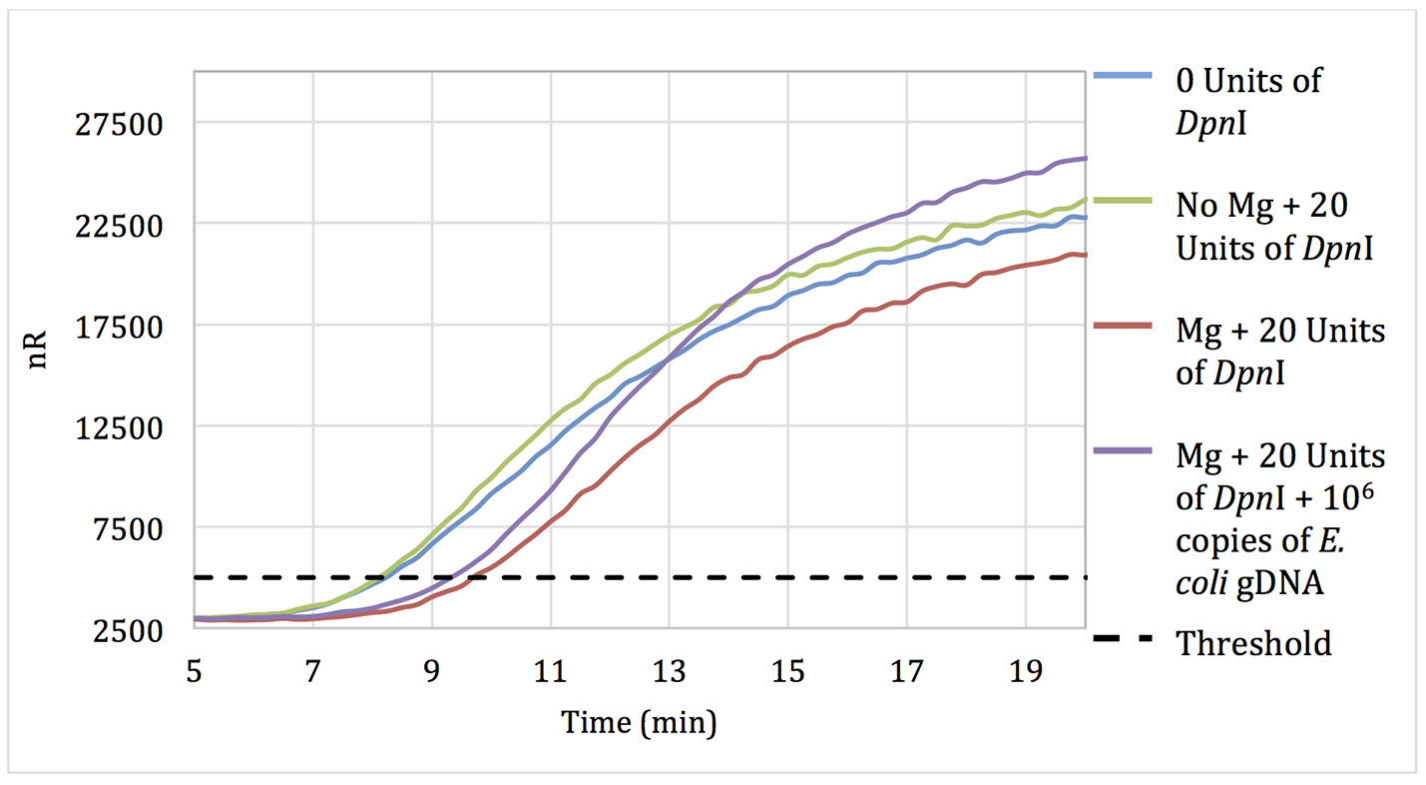
Amplification curves of RPA reactions carried out using primer set 1 and untreated reagents (blue) or reagents pre-treated with 20 units of DpnI for 15 min (red). Reagents pre-treated with DpnI and spiked with 10^6^ copies of *E. coli* gDNA (purple); reagents pre-treated with DpnI in the absence of MgAc (green).

No-template RPA reactions that contained pellets pre-treated for 15 min with 20 units of DpnI showed only a 90-s delay in threshold time when compared with reactions using untreated pellets, suggesting that some but not all of the extraneous DNA was digested during DpnI treatment (Figure 1). Negative control reactions with pellets incubated with DpnI in the absence of magnesium (DpnI's co-factor) showed the same threshold time as reactions using untreated RPA reagent.

Increasing the DpnI digestion time from 15 to 60 min resulted in no increase in the threshold time difference between no-template reactions with treated and untreated RPA reagent. However, increasing the DpnI concentration 5- or 7-fold resulted in a 2.75 and 5.5-min increases in the threshold time, respectively (data not shown). Results indicate that treating RPA reagents with DpnI can digest and render a significant proportion of contaminating DNA un-amplifiable in subsequent applications.

#### DNA depletion using anti-DNA antibodies

**Figure 2:**
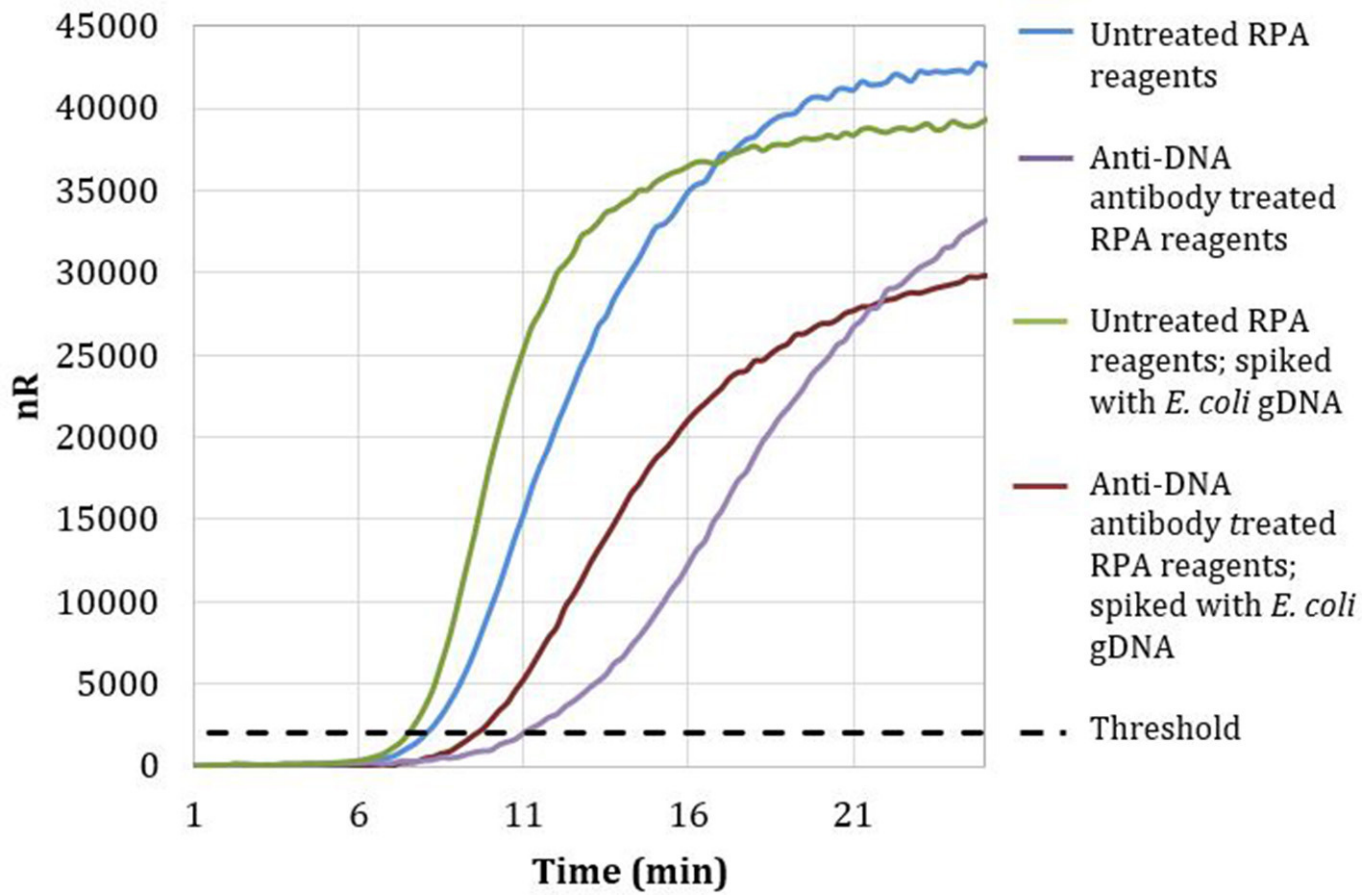
Amplification curves of RPA reactions carried out with RPA primer set 1 and untreated (blue) or treated (purple) reagents. Untreated reactions spiked with 100 copies of *E. coli* gDNA (green) exhibited a threshold time of 7.25 min, whereas anti-DNA antibody treated reactions spiked with 100 copies of *E. coli* gDNA (maroon) exhibited a threshold time of 9.5 min.

Magnetic particles conjugated with anti-DNA antibodies with affinity for both dsDNA and ssDNA were used to treat RPA reagents, as described in section Materials and Methods. No-template RPA reactions that were pretreated with anti-dsDNA antibody magnetic particles showed a 3-min later threshold time than reactions involving untreated reagents (Figure 2). These data demonstrate that treating RPA reagents with anti-dsDNA antibody magnetic particles removes a significant proportion of extraneous DNA.

Reactions with treated reagents showed a 1.75-min earlier threshold time when spiked with 100 copies of *E. coli* gDNA compared with reactions with treated reagents in the absence of spiked *E. coli* gDNA (Figure 2). These data indicate that treatment with anti-dsDNA antibody magnetic particles does not significantly remove proteins necessary for RPA amplification.

Both DpnI treatment and anti-dsDNA antibody magnetic particles effectively removed extraneous DNA, with similar efficiencies, from RPA reagents. Treating RPA reagents with 100 units of DpnI showed a 2.75 min delay in threshold time when compared to reactions with untreated reagents. RPA reagents treated with anti-dsDNA antibody showed a comparable delay of 3 min in threshold time when compared to reactions with untreated reagents.

The cost of RPA reagents is ~$5 per reaction; these reagents can be treated to remove extraneous DNA with 100 units of DpnI at a cost of $3.33, or using anti-DNA antibody and magnetic particles for $2.25 per reaction. Thus later work employed the slightly less-expensive anti-DNA magnetic particles.

### Quantitative bacterial detection using RPA

**Figure 3:**
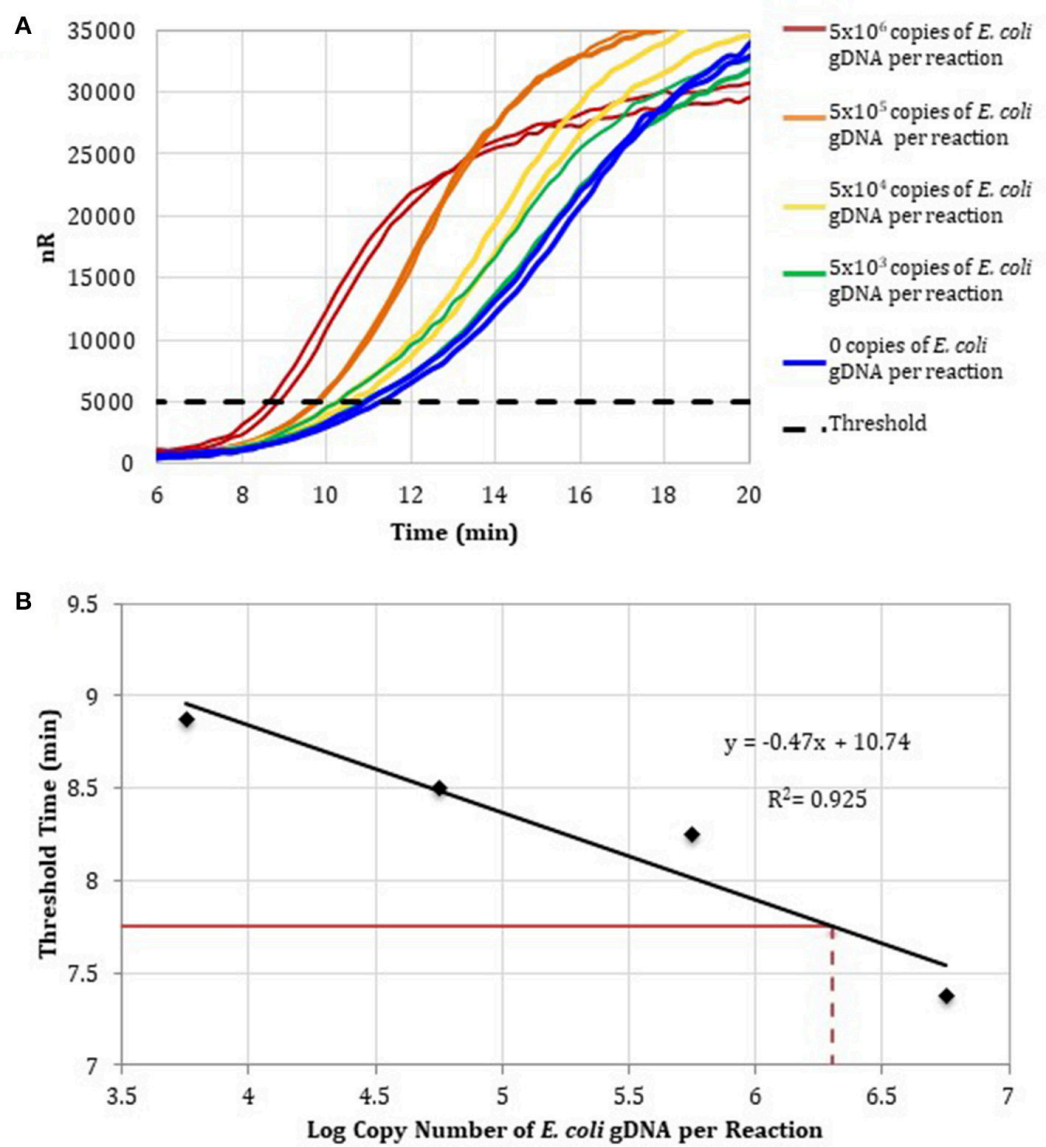
**(A)** RPA amplification curves generated using the primer set 1, 0–10^6^ copies of *E. coli* gDNA (2 μL per reaction; *n* = 2), and RPA reagents pre-treated with anti-dsDNA antibody-coupled magnetic particles. **(B)** RPA standard curve was generated using the average threshold times calculated from measurements in **(A)** (*n* = 2). The fecal DNA exhibited an average threshold time of 7. 75 min (solid red line) and an estimated load of 6.3-log copies of bacterial gDNA per reaction (dotted red line) or a total bacterial load of 1.01 × 10^7^ bacterial gDNA copies per 15 ng of isolated gDNA.

To determine the detection range of the assay based on DNA-depleted reagents, 10-fold serial dilutions of *E. coli* gDNA standards were spiked into RPA reactions containing RPA reagents pre-treated with anti-ds DNA antibody magnetic particles (Figure 3A). Two replicates for concentrations ranging from 10^6^ to 10^3^ *E. coli* gDNA copies per reaction and two no-template control reactions were performed. Threshold time versus log copy number of *E. coli* gDNA was fit to a semi-logarithmic regression line to generate a standard curve (Figure 3B).

To demonstrate that the total bacterial load in a clinically relevant sample can be quantified using RPA, DNA was isolated from a human fecal sample (Analytical Biological Services, Inc.) and amplified in RPA reactions. Based on the *E. coli* standard curve, the total bacterial load of the fecal sample was 1.01 × 10^7^ bacterial gDNA copies per 15 ng of isolated gDNA.

The same fecal sample was also re-analyzed using real-time PCR. A total of 2 μL of each *E. coli* gDNA standard dilution was spiked into PCR reactions containing previously published pan bacteria–specific primers (Table 1, primer set 2; *n* = 3). Threshold cycle was plotted against log copies of *E. coli* gDNA per reaction to generate a standard curve (Figure S4), which was then used to calculate the total bacterial load of the fecal sample as 5.58 × 10^6^ bacterial gDNA copies per 15 ng of isolated DNA. The total bacterial load of the stool sample quantified by qPCR differed by less than two-fold from the total bacterial load estimated by quantitative RPA (1.01 × 10^7^ bacterial gDNA copies per 15 ng of isolated gDNA).

### Development of the *A. muciniphila* assay

#### Absolute *A. muciniphila* abundance

**Figure 4:**
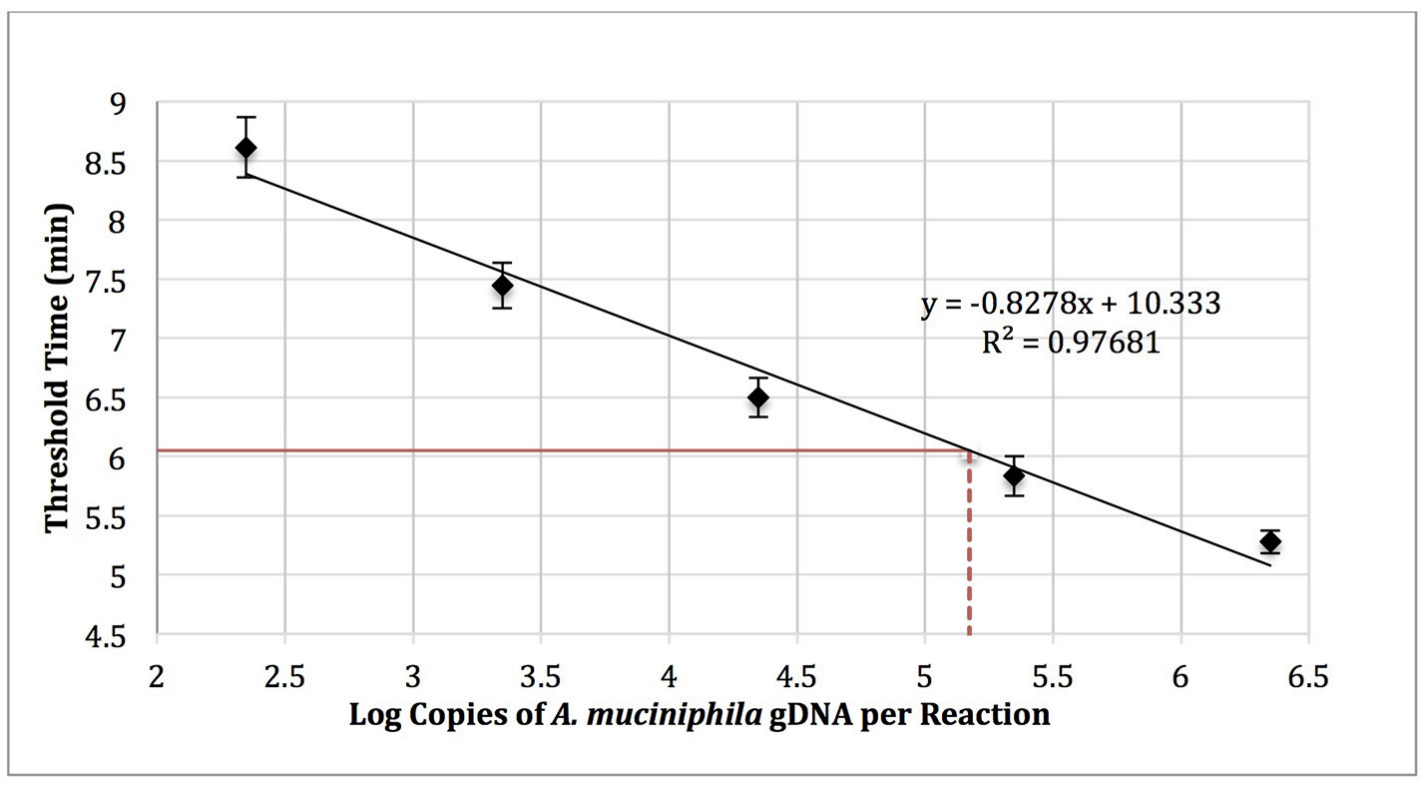
RPA standard curve of *A. muciniphila* gDNA (0-1,000,000 copies; ATCC BAA-835) amplified using real-time SYBR green-RPA and primer set 1 (*n* = 3). The average threshold time of a 10-fold dilution of the fecal sample gDNA was 6.05 ± 0.1 min (solid red line, *n* = 3). The estimated load was 1.48 × 10^5^ copies of *A. muciniphila* gDNA per reaction (dotted red line) or 2.99 × 10^5^ copies of *A. muciniphila* gDNA per 15 ng of DNA isolated from the fecal sample.

*Akkermansia muciniphila* gDNA (0–1,000,000 copies; ATCC BAA-835) was amplified using real-time SYBR Green-RPA with *A. muciniphila*–specific primers designed in this work (Table 1, primer set 3). The amplification curves were used to determine threshold time values and generate a standard curve (log copy number gDNA vs. threshold cycle, Figure 4). Reactions run with no added template (NTC) exhibited an average threshold time of 10.8 ± 0.42 min (*n* = 3). Reactions spiked with 1,000 copies *E. coli* gDNA (ATCC 35218) exhibited an average threshold time insignificantly different from that of the NTC (10.6 min; *n* = 3).

Five microliters of a 10-fold dilution of gDNA isolated from the fecal sample (15 ng gDNA per μl) was run as a template in RPA reactions to yield an average threshold time of 6.05 ± 0.1 min (*n* = 3). The standard curve (Figure 4) was then used to quantify the absolute load of *A. muciniphila* as 2.99 × 10^5^ gDNA copies per 15 ng of DNA isolated from the fecal sample.

The absolute *A. muciniphila* concentration in the fecal sample was separately determined by qPCR using primer set 4 (Table 1), which reportedly enables determination of the absolute abundance of *A. muciniphila* via qPCR (Collado et al., 2007; Schneeberger et al., 2015; Guo et al., 2016). The absolute *A. muciniphila* load of the fecal sample was estimated based on the qPCR semi-logarithmic regression line as 8.91 × 10^4^ gDNA copies per reaction, or 1.78 × 10^5^ gDNA copies per 15 ng of isolated gDNA (Figure S5), quite similar to the absolute *A. muciniphila* load of the fecal sample as determined using RPA (2.99 × 10^5^ bacterial gDNA copies per 15 ng of isolated gDNA).

### Relative *A. muciniphila* abundance

Relative *A. muciniphila* abundance was calculated as the ratio of *A. muciniphila* 16S copies to total bacterial 16S copies using both qPCR (using primer sets 2 and 4) and RPA (using primer sets 1 and 3) to show a relative abundance of 1.36 and 1.29%, respectively. The relative *A. muciniphila* abundance of the fecal sample was determined using RPA from 3 × 2.99 × 10^5^ *A. muciniphila* 16S copies per 15 ng of gDNA divided by 7 × 1.01 × 10^7^ bacterial 16S copies per 15 ng of gDNA) and from PCR 5.34 × 10^5^ *A. muciniphila* 16S copies/3.91 × 10^7^ bacterial 16S copies) as 3 × 1.78 × 10^5^ *A. muciniphila* 16S copies per 15 ng of gDNA divided by 7 × 5.58 × 10^6^ bacterial 16S copies per 15 ng of gDNA.

In 16S rRNA gene sequencing, relative *A. muciniphila* abundance was defined as the ratio of *A. muciniphila* reads to the total number of bacterial reads. In 16S rRNA sequencing, the fecal sample produced 14,358,714 reads, of which 14,285,134 (99.5%) were taxonomically classified as bacteria. A total of 297,040 reads (2.07% of all reads) were classified as *A. muciniphila* ATCC BAA-835–specific, in good agreement with the RPA result.

When compared to sequencing, RPA gave a slightly lower relative *A. muciniphila* abundance in the fecal sample. This result could have been due to off-target amplification of the bacteria-specific primers. Note that the accuracy of the relative *A. muciniphila* abundance RPA assay, as with all nucleic acid-based assays, is highly dependent upon the quality of the primers. RPA assay sensitivity could perhaps be improved by increasing the primer specificity.

## Discussion

Many microbial taxonomic groups have been identified as beneficial, detrimental, or simply indicative of a wide range of health conditions. As the number of organisms of interest expands, so does the need for inexpensive, accurate, and quantitative measurement of bacterial abundance within the gut. Unlike qPCR or 16S sequencing, RPA enables field-based testing. Recombinase polymerase amplification (RPA) is an isothermal nucleic acid amplification method that employs commercially available, easy-to-use freeze-dried, reaction-ready enzyme pellets that can be used to analyze specimens rapidly in the field, using a portable fluorometer. Analyzing complex microbial communities immediately after sampling can lead to a more accurate quantification of relative bacterial abundance. RPA is a method that is quickly growing in usage and range of applications (Piepenburg et al., 2008; Kim and Easley, 2011; Loo et al., 2013; Shin et al., 2013; Xu et al., 2014; Tortajada-Genaro et al., 2015; Daher et al., 2016; Yamanaka et al., 2017).

While many groups have developed RPA assays that demonstrate species-specific detection (Euler et al., 2012b; Ahmed et al., 2014; Krõlov et al., 2014; Clancy et al., 2015; Liljander et al., 2015; Cabada et al., 2017), there has been no development of an RPA assay that performs bacterial-specific detection. This gap is due to a high level of *E. coli* DNA in commercial RPA reagents. This paper is the first to offer a solution to this contamination. After successfully removing the contaminating DNA, we proceeded to demonstrate successful RPA quantification of bacteria in a stool sample.

In this proof-of-concept study we enabled the estimation of the relative abundance of *A. muciniphila* in human fecal DNA and demonstrated the promise of RPA as an inexpensive and accurate tool for measuring gut microbiome marker organisms. *A. muciniphila* appears to play a pivotal role in insulin resistance and inflammation. Furthermore, low abundance of *A. muciniphila* in the human gut is found to correlate with obesity. More recent studies indicate that *A. muciniphila* can influence the effectiveness of certain cancer immunotherapy drugs (Gopalakrishnan et al., 2018; Routy et al., 2018). Altering patients' gut microbiota to be rich in *A. muciniphila* may increase the fraction of individuals who respond to cancer immunotherapies. Given the short time *A. muciniphila* has been studied, the multitude of studies that support its role as a beneficial bacterium suggests its important role in human health. The medical community may soon need an inexpensive screening tool for identifying individuals with low fecal *A. muciniphila* abundance. To further assess the analytical accuracy and sensitivity of *Akkermansia muciniphila* detection with RPA more fecal samples should be tested.

## Author contributions

HG helped conceive the project, designed and executed experiments, and wrote the manuscript. DC helped to design and perform experiments, and edited the manuscript. MC helped to interpret and conceive the project, and edited the manuscript. KK interpreted experiments, and helped to write and edit the manuscript. RW conceived the project, interpreted experiments, and helped to write and edit the manuscript.

### Conflict of interest statement

The authors declare that the research was conducted in the absence of any commercial or financial relationships that could be construed as a potential conflict of interest. The reviewer JG and handling Editor declared their shared affiliation.
