# Corrigendum: *Akkermansia muciniphila* as a Model Case for the Development of an Improved Quantitative RPA Microbiome Assay

**DOI:** 10.3389/fcimb.2019.00032

**Published:** 2019-03-21

**Authors:** Heather J. Goux, Dimple Chavan, Mary Crum, Katerina Kourentzi, Richard C. Willson

**Affiliations:** ^1^Department of Biology and Biochemistry, University of Houston, Houston, TX, United States; ^2^Department of Chemical and Biomolecular Engineering, University of Houston, Houston, TX, United States; ^3^Tecnológico de Monterrey-ITESM Campus Monterrey, Monterrey, Mexico

**Keywords:** RPA, gut microbiome, *Akkermansia muciniphila*, bacterial quantification, point-of-need

In the original article, there was an error. The reported relative *A. muciniphila* abundance measurements failed to sufficiently take into account that variable numbers of copies of the 16S rRNA gene can occur in a bacterial genome. The *E. coli* or *A. muciniphila* gDNA copies per mass of isolated DNA were determined from the threshold time (RPA) or cycle (PCR) and the appropriate standard curves. After this step, the resulting gDNA copies were not multiplied by the number of 16S rRNA genes that occur per genome of the standard. The number of *A. muciniphila* gDNA copies should have been multiplied by three, and the number of *E. coli* gDNA copies should have been multiplied by seven. Only then can the relative *A. muciniphila* abundance be calculated as the ratio of *A. muciniphila* 16S gene copies to the number of bacterial 16S copies.

A correction has been made to **Materials and Methods**, section **Determining Total Bacterial and** ***A. muciniphila*** **Abundance**. The following paragraph has been added:

For both *A. muciniphila* (ATCC BAA-835) and *E. coli* (ATCC 35218) gDNA standards, the 16S rRNA gene sequences from the GenBank (accession nos. NR_074436.1 and EF436579, respectively) were aligned to the complete genome sequences of *A. muciniphila* (accession no. NC_010655.1) and *E. coli* (accession NZ_KK583188.1) using the NCBI BLAST. This resulted in three and seven matches (100% in identity and composition) for *A. muciniphila* and *E. coli*, respectively. Thus, to calculate the 16S rRNA gene copies for each standard, the genomic DNA copies were multiplied by the number of 16rRNA gene copies per genome (3 for *A. muciniphila* and 7 for *E. coli*).

A correction has been made to **Results**, section **Development of the** ***A. muciniphila*** **Assay**, subsection **Absolute** ***A. muciniphila*** **Abundance**, paragraph three. The updated paragraph reads as follows:

The absolute *A. muciniphila* load of the fecal sample was estimated based on the qPCR semi-logarithmic regression line as 8.91 × 10^4^ gDNA copies per reaction, or 1.78 × 10^5^ gDNA copies per 15 ng of isolated gDNA (Figure S5), quite similar to the absolute *A. muciniphila* load of the fecal sample as determined using RPA (2.99 × 10^5^ bacterial gDNA copies per 15 ng of isolated gDNA).

Corrections have also been made to **Results**, section **Development of the** ***A. muciniphila*** **Assay**, subsection **Relative** ***A. muciniphila*** **Abundance**, paragraphs one and three. The updated paragraph one reads as follows:

Relative *A. muciniphila* abundance was calculated as the ratio of *A. muciniphila* 16S copies to total bacterial 16S copies using both qPCR (using primer sets 2 and 4) and RPA (using primer sets 1 and 3) to show a relative abundance of 1.36 and 1.29%, respectively. The relative *A. muciniphila* abundance of the fecal sample was determined using RPA from 3 × 2.99 × 10^5^ *A. muciniphila* 16S copies per 15 ng of gDNA divided by 7 × 1.01 × 10^7^ bacterial 16S copies per 15 ng of gDNA) and from PCR 5.34 × 10^5^ *A. muciniphila* 16S copies/3.91 × 10^7^ bacterial 16S copies) as 3 × 1.78 × 10^5^ *A. muciniphila* 16S copies per 15 ng of gDNA divided by 7 × 5.58 × 10^6^ bacterial 16S copies per 15 ng of gDNA.

The updated paragraph three reads as follows:

When compared to sequencing, RPA gave a slightly lower relative *A. muciniphila* abundance in the fecal sample. This result could have been due to off-target amplification of the bacteria-specific primers. Note that the accuracy of the relative *A. muciniphila* abundance RPA assay, as with all nucleic acid-based assays, is highly dependent upon the quality of the primers. RPA assay sensitivity could perhaps be improved by increasing the primer specificity.

The authors apologize for these errors and state that this does not change the scientific conclusions of the article in any way. The original article has been updated.

