# Supplementary material for: Akkermansia muciniphila as a Model Case for the Development of an Improved Quantitative RPA Microbiome Assay
Source: Front Cell Infect Microbiol. 2018 Jul 12;8:237. doi: 10.3389/fcimb.2018.00237 (PMC6052657; doi:10.3389/fcimb.2018.00237)
Supplement: Supplementary file 1 [file Image_1.PDF]

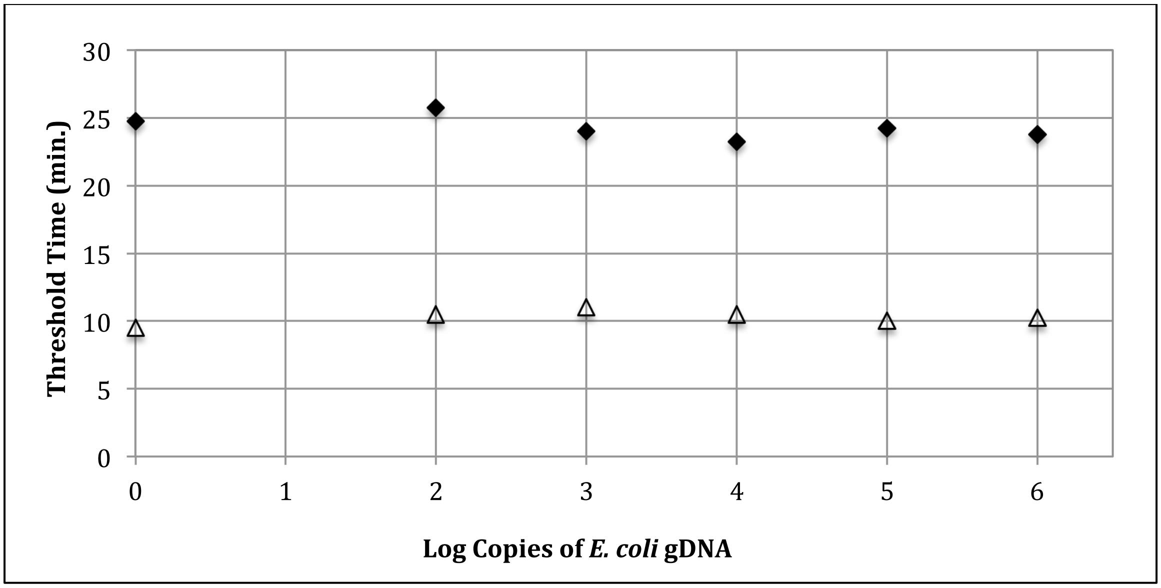


**Figure S1**. Real-time RPA reactions run with unprocessed RPA reagents, 10-fold dilutions of *E. coli* gDNA *(*ATCC 35218*)*, and newly designed (Primer Set 1) or previously reported (Primer Set 2) bacteria specific primer pairs. Data points in series 1 represent reactions run with Primer Set 1 (Δ) and only shows a 90 sec standard deviation in threshold time (range: 9.5 – 11 min; n=1). Data series 2 represents reactions run with Primer Set 2 (u) and has a standard deviation of 150 sec in threshold time (range: 23.25 min – 25.75 min; n=1).
