# Supplementary material for: Akkermansia muciniphila as a Model Case for the Development of an Improved Quantitative RPA Microbiome Assay
Source: Front Cell Infect Microbiol. 2018 Jul 12;8:237. doi: 10.3389/fcimb.2018.00237 (PMC6052657; doi:10.3389/fcimb.2018.00237)
Supplement: Supplementary file 2 [file Image_2.PDF]

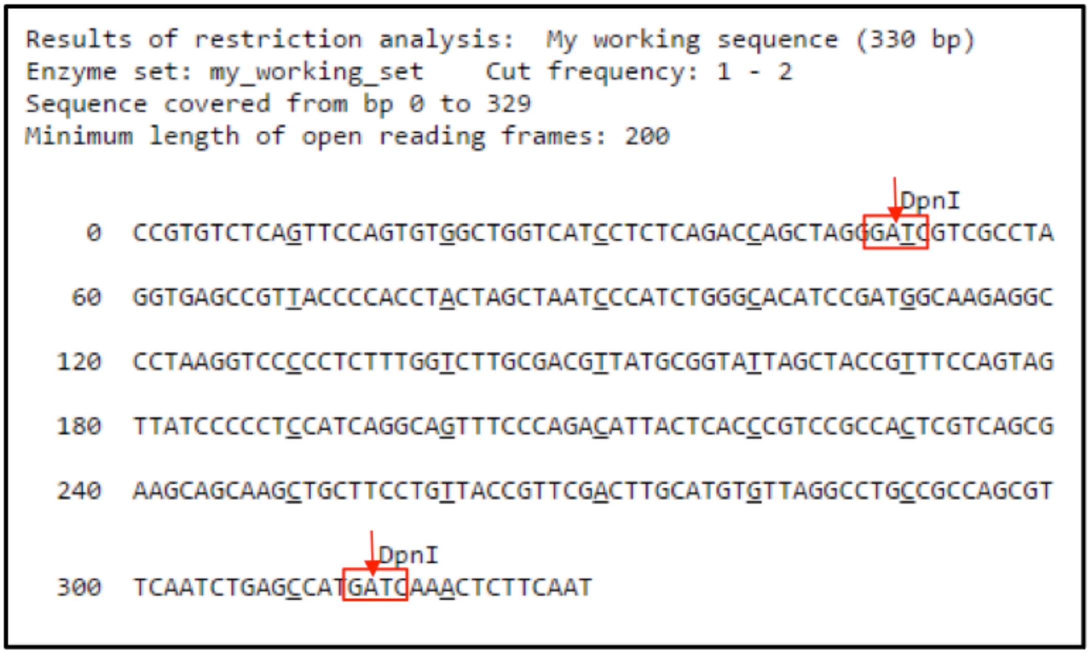

Figure S2. *Dpn*I restriction enzyme cut sites (red boxes with arrows) located within the previously sequenced amplicons generated from no-template control reactions. The image was obtained from WatCut, an online restriction analysis tool (http://watcut.uwaterloo.ca/template.php).
