# Supplementary material for: Akkermansia muciniphila as a Model Case for the Development of an Improved Quantitative RPA Microbiome Assay
Source: Front Cell Infect Microbiol. 2018 Jul 12;8:237. doi: 10.3389/fcimb.2018.00237 (PMC6052657; doi:10.3389/fcimb.2018.00237)
Supplement: Supplementary file 3 [file Image_3.PDF]

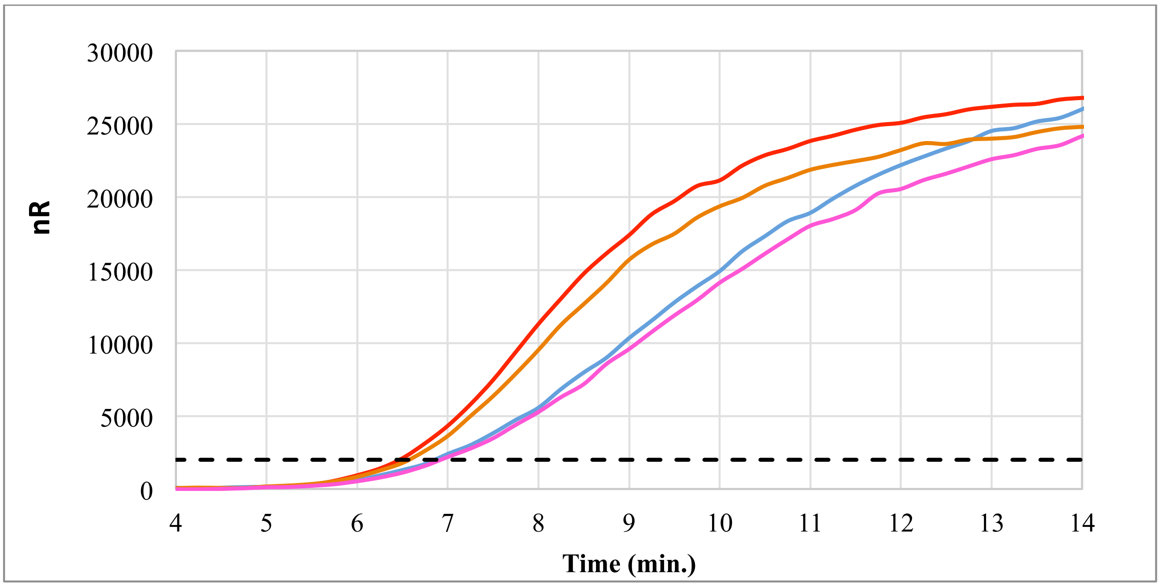

Figure S3. Amplification curves of no-template RPA reactions run with Primer Set 1 and sonicated (red and orange) or un-sonicated (pink and blue) reaction pellets (n=2 each). Reactions that contained sonicated pellets met the threshold fluorescence (dashed line) at 6.25 and 6.5 minutes. No template control reactions with non-treated pellets reached the threshold fluorescence at 7.25 and 7.5 minutes.
