# Supplementary material for: Akkermansia muciniphila as a Model Case for the Development of an Improved Quantitative RPA Microbiome Assay
Source: Front Cell Infect Microbiol. 2018 Jul 12;8:237. doi: 10.3389/fcimb.2018.00237 (PMC6052657; doi:10.3389/fcimb.2018.00237)
Supplement: Supplementary file 4 [file Image_4.PDF]

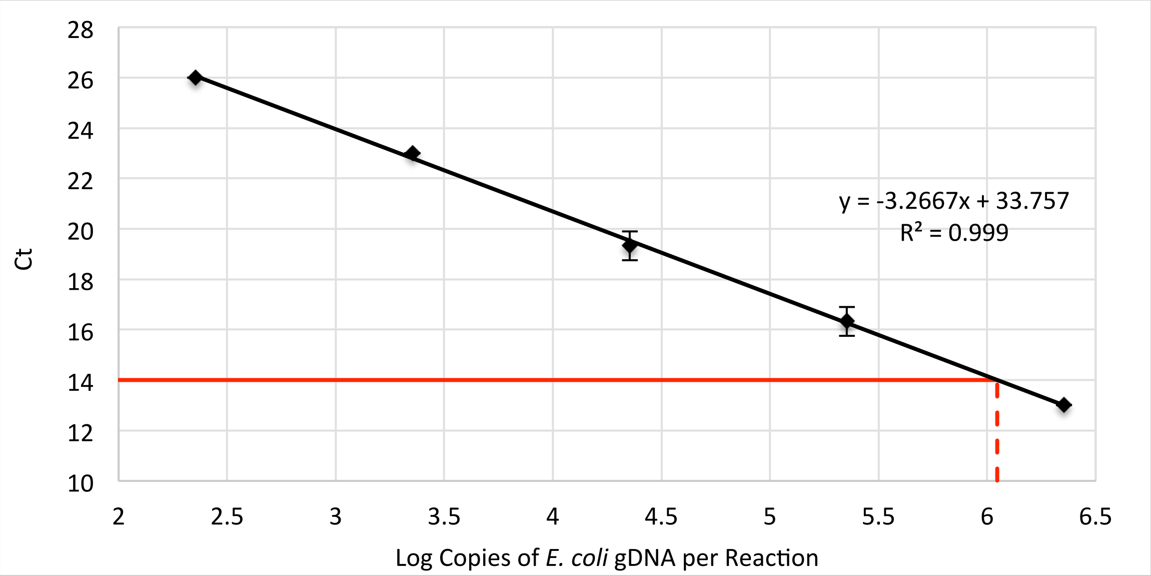

Figure S4. A semi-log regression line generated from amplification curves of PCR reactions run with Primer Set 2 and dilutions of E. coli gDNA (0 – 10^6 copies per µl; 2 µl per reaction) (threshold cycle vs. Copies of *E. coli* gDNA per reaction; n=3). The semi-log regression line and the average threshold time of PCR reactions run with isolated gDNA from stool (2 µl of a 10-fold dilution of the stock concentration; 3 ng) to determine a total bacterial load of 1.12 x 10^6 bacterial gDNA copies per reaction (5.58 x 10^6 bacterial gDNA copies per 15 ng of isolated gDNA).
