# Supplementary material for: Akkermansia muciniphila as a Model Case for the Development of an Improved Quantitative RPA Microbiome Assay
Source: Front Cell Infect Microbiol. 2018 Jul 12;8:237. doi: 10.3389/fcimb.2018.00237 (PMC6052657; doi:10.3389/fcimb.2018.00237)
Supplement: Supplementary file 5 [file Image_5.PDF]

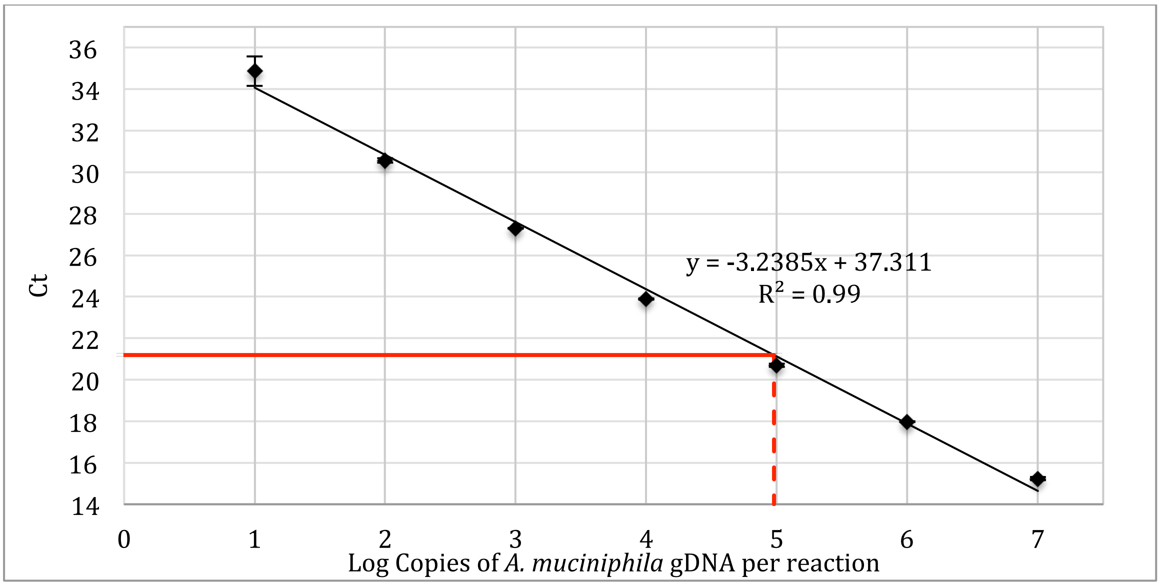

Figure S5 Semi-log regression line of reactions with ten-fold dilutions of *Akkermansia muciniphila* genomic DNA (10 – 10^7 copies; ATCC– BAA-835*)* amplified using Realtime-SYBR Green-PCR and Primer Set 4 (n=3). 7.5 ng of isolated stool DNA (5 µl of a 10-fold dilution of the stock concentration) was run as template in PCR reactions to yield a Ct of 21.19. The standard curve was used to calculate the absolute *A. muciniphila* abundance, 8.91 x 10^4 copies *A. muciniphila* gDNA per reaction (1.78 x 10^5 *A. muciniphila* gDNA copies per 15 ng of isolated gDNA). Both no-template reactions and negative control reactions (reactions with was 2,000 copies of *E. coli* gDNA as template) yielded a threshold cycle of 38.
